# Does Loneliness Predict Subsequent Use of Flu Vaccination? Findings from a Nationally Representative Study of Older Adults in Germany

**DOI:** 10.3390/ijerph16244978

**Published:** 2019-12-07

**Authors:** André Hajek, Hans-Helmut König

**Affiliations:** Department of Health Economics and Health Services Research, University Medical Center Hamburg-Eppendorf, 20246 Hamburg, Germany; h.koenig@uke.de

**Keywords:** vaccination, loneliness, flu shot, social relationship, influenza

## Abstract

There is a lack of studies investigating whether loneliness predicts subsequent use of flu vaccination. Therefore, we aimed to clarify this relationship. Data were drawn from two waves (second wave took place in 2002, third wave took place in 2008) of a nationally representative cohort of community-dwelling individuals in Germany. The sample was restricted to individuals ≥60 years for whom flu vaccination is recommended. Loneliness was quantified using the De Jong Gierveld Loneliness Scale (second wave). Flu vaccination in the past 12 months was assessed (third wave). Consequently, older individuals that participated in the second wave and reported flu vaccination in the third wave were included (*n* = 970). The other waves (e.g., first wave) were excluded for reasons of data availability. Increased loneliness was associated with subsequent decreased use of the flu vaccine. Moreover, the probability of flu vaccination in the third wave was positively associated with being retired (ref.: employed), having a lower income, and the number of physical illnesses in the second wave. Findings stressed the importance of loneliness in the decreased use of the flu vaccine. Preventing loneliness may also help to increase flu vaccination rates.

## 1. Introduction

Influenza (seasonal flu) is an infectious disease that can have severe health consequences, particularly for vulnerable groups such as pregnant women, children, and individuals in old age. Globally, it causes 300,000 to 500,000 deaths per year [1]. Vaccination is an effective prevention for seasonal flu. This has been demonstrated among pregnant women and individuals in old age [2]. 

Vaccination is not compulsory in Germany; however, the German Standing Committee on Vaccination (STIKO) recommends flu vaccination for pregnant women, individuals with certain chronic illnesses, health care workers, individuals residing in nursing homes, and individuals aged 60 and above [3]. Effective vaccination should be repeated every year because the virus pathogen changes over time. A common place to get a flu shot is the practice of a general practitioner (GP). If recommended, the costs of vaccination are fully reimbursed by sickness funds. Depending on the statutes of their health insurance, flu vaccination is also free of charge for other patients. 

As taking a flu shot is recommended, but not compulsory, for certain groups, there are numerous studies that investigate the determinants of flu vaccination, particularly for the risk groups listed above. There are systematic reviews focusing on socioeconomic determinants of flu vaccination among individuals in old age [4]. Drawing on the Health Belief Model, other factors such as illness-specific self-efficacy [5], risk assessment [6], or feelings about flu vaccination [7] have also been studied. Consequently, previous studies examining psychosocial factors have been mainly restricted to perceptions or beliefs directly related to flu vaccination. Adopting a broader perspective, one recent study investigated whether more general psychosocial factors, such as optimism or subjective well-being, were associated with flu vaccination, using data from a nationally representative sample [8]. They found that self-esteem and perceived stress were associated with flu vaccination in multiple regression models. However, there is a lack of studies analyzing whether loneliness (discrepancy between the need and the extent to which the social need is satisfied by means of meaningful social interactions [9]) predicts subsequent flu vaccination. Based on a large, nationally representative sample of older adults, the aim of this study was to examine whether loneliness is associated with subsequent use of the flu vaccine. This study may contribute to our understanding about the factors related to flu vaccination in individuals aged 60 and above [10].

Since it has been demonstrated that loneliness is inversely related to the use of preventive health care services (e.g., cancer screening [11,12]), we hypothesized that decreased loneliness is related to increased use of the flu vaccine among individuals ≥60 years. 

## 2. Materials and Methods 

### 2.1. Sample

For this study, data were drawn from the nationally representative German Ageing Survey (DEAS) covering individuals ≥40 years (second half of life) living in private households. Topics of the survey included perception of ageing, labor force participation, health, and subjective well-being. The first wave took place in 1996. Further waves took place in 2002 (second wave), 2008 (third wave), 2011 (fourth wave), 2014 (fifth wave), and 2017 (sixth wave). The DEAS study has a cohort-sequential design (i.e., consisting of new baseline samples that were introduced every six years (2002, 2008, 2014) and additional panel samples that were introduced in 2011 and 2017). While 5194 individuals were interviewed in the second wave (38% response rate), 8200 individuals took part in the third wave (38% response rate). It has been demonstrated that the DEAS study has similar response rates to other survey studies conducted in Germany [13]. Additional details with regard to the DEAS study have been provided by Klaus et al. [14].

We restricted our sample to the second and third waves because meaning in life was first assessed in 2002. Thus, individuals were included who participated in the second wave and reported flu vaccination in the third wave (*n* = 970). Furthermore, our sample was restricted to individuals aged 60 and over, as the STIKO recommends yearly flu vaccination for this age group.

Written informed consent was given by all individuals. An ethical statement for the DEAS study was not needed because the criteria for it were not met (e.g., using invasive methods, risk for the respondents, and examination of patients). 

### 2.2. Outcome Measure

Self-reported flu vaccination in the last 12 months (yes, no) was used as the outcome measure. Bhandari et al. [15] showed that this recall period was appropriate for assessing health care utilization. 

### 2.3. Independent Variables

Loneliness was quantified using the 6-item version (four levels) of the Gierveld and van Tilburg scale [16]. The score was computed by averaging the responses to the 6 items. In this study, Cronbach’s alpha was 0.83. Other studies have demonstrated that this scale has favorable psychometric properties [17].

Based on previous studies as well as theoretical considerations, gender, age, family status (married, living together; married, living separated; divorced; single; widowed), labor force participation (employed; retired; other: not employed), income (log household net equivalent income), and meaning in life (To what extent do you feel your life to be meaningful? 1 = not at all; 2 = a little; 3 = a moderate amount; 4 = very much; 5 = extremely) were adjusted for in the analysis. 

Self-rated health (ranging from 1 = very good to 5 = very bad), physical functioning (subscale physical functioning of the Short Form 36 (SF-36); [18] from 0 = worst to 100 = best), depressive symptoms (15 item version of the Center for Epidemiological Studies Depression Scale (CES-D), [19] from 0 to 45, CES-D ≥ 18 indicated depression), as well as the number of chronic illnesses such as cancer (ranging from 0 to 11) were also adjusted for in the analysis. 

### 2.4. Statistical Analysis

First, sample characteristics stratified by individuals who were vaccinated in the past 12 months and those who were not (wave 3) were reported. Second, multiple logistic regressions were computed to analyze the association between loneliness and flu vaccination. The 0.05 level of probability was adopted as the criterion for statistical significance. Stata 15.1 was used for analysis. 

## 3. Results

### 3.1. Bivariate Analysis

Sample characteristics stratified by individuals that were vaccinated in the past 12 months and those that were not (wave 3) are depicted in Table 1. While the average loneliness score (wave 2) among individuals that were vaccinated in the subsequent wave 3 was 1.6 (±0.5), the average score was 1.7 (±0.5) among those not vaccinated. Moreover, those vaccinated reported a higher number of physical illnesses (2.8 ± 1.7) and worse physical functioning (83.9 ± 19.9) compared to those who were not (physical illnesses: 2.2 ± 1.7; physical functioning: 87.3 ± 18.6). Further details are displayed in Table 1. 

### 3.2. Regression Analysis

Findings of multiple logistic regressions are displayed in Table 2. Regressions showed that loneliness was associated with subsequent decreased use of the flu vaccine (OR: 0.71, 95% CI: 0.51–0.97). Furthermore, the likelihood of being vaccinated in the third wave was positively associated with being retired (ref.: employed; OR: 1.99, 1.23–3.23), lower log household net equivalent income (OR: 0.62, 0.44–0.88), and the number of physical illnesses (OR: 1.20, 1.09–1.32) in the second wave. Use of the flu vaccine in the third wave was not associated with sex, age, family status, meaning in life, depression, self-rated health, and physical functioning in the second wave. 

## 4. Discussion

We aimed to clarify whether loneliness predicts subsequent use of the flu vaccine based on a nationally representative sample of individuals ≥60 years in Germany. This is important because loneliness is common in old age (e.g., due to the loss of a spouse, friends, and acquaintances, or due to the loss of functional abilities or admission to a nursing home) and is associated with various adverse health outcomes. Furthermore, influenza is associated with subsequent mortality [1], and vaccination is an effective prevention for the seasonal flu [2]. 

In our study, multiple logistic regressions revealed that loneliness was associated with a subsequent decreased use of the flu vaccine. The probability of flu vaccination in the third wave was positively associated with being retired, lower income, and number of physical illnesses. However, it was not associated with sex, age, family status, meaning in life, depression, self-rated health, and physical functioning. 

Loneliness is commonly associated with adverse health outcomes (e.g., worse mental or physical health). This was also the case in our study. Thus, intuitively one may assume that the link between loneliness and subsequent decreased use of the flu vaccine can mainly be explained by health-related factors. However, our study adjusted for various important health-related factors (depression, self-rated health, physical functioning, and the number of chronic diseases) in regression analysis. With regard to other alternative explanations (e.g., marital status or lack of meaning in life), it is worth noting that our study also adjusted for several socioeconomic factors and meaning in life in regression analysis. 

To the best of our knowledge, no studies have been conducted that examine the link between loneliness (using validated instruments) and subsequent flu vaccination. Therefore, it is difficult to compare our study with previous studies. However, some studies have shown that loneliness was negatively associated with the use of other preventive health care services such as cancer screening [11,12]. These associations ((i) loneliness and cancer screening as well as (ii) loneliness and flu vaccination in our study) may be explained by the fact that the use of these preventive health care services was not perceived as the social norm among individuals scoring high in loneliness, as they may not have perceived social pressure to use these services by family or friends [20]. Furthermore, individuals scoring high in loneliness may have had a general lack of motivation to use preventive health care services. Nevertheless, future research is required to clarify the underlying mechanisms. 

With regard to the strengths and limitations of this study, it is worth noting that a nationally representative sample of older adults living in the community was used. Furthermore, loneliness was measured using an established scale. Two waves were used. Various potential confounders were adjusted for in the analysis. With regard to limitations, self-reports were used in this study, and a small selection bias was detected in the DEAS study.

## 5. Conclusions

Aside from numerous other negative consequences of loneliness reported in previous studies, it is also associated with subsequent decreased use of the flu vaccine. Preventing loneliness may also help to increase flu vaccination rates.

This is the first study determining the link between loneliness and subsequent flu vaccination in older adults. We hope that our work will inspire future work in this area. More specifically, in future research, it may be worth investigating the link between loneliness and flu vaccination in other vulnerable groups, such as pregnant women, medical staff, or, in particular, in individuals with chronic diseases (e.g., metabolic diseases such as diabetes, chronic obstructive pulmonary disease, liver or kidney diseases; or chronic neurological diseases such as multiple sclerosis). Furthermore, this link should be investigated in other cultural settings because it appears plausible that the association between loneliness and flu vaccination varies across cultures. 

## Figures and Tables

**Table 1:** Sample characteristics stratified by use of flu vaccination.

	Persons Who Had a Flu Shot in the Past 12 Months (Assessed at Wave 3) (430; 44.3%)		Persons Who Did Not Have a Flu Shot in the Past 12 Months (Assessed at Wave 3) (540; 55.7%)		*p*-Value
Independent variables (assessed at wave 2)	N/Mean	%/SD	N/Mean	%/SD	
Gender: female	266	58.1%	192	41.9%	0.15
Age in years	66.5	7.1	64.3	7.7	<0.001
Marital status					
Married and living together with spouse	419	56.9%	318	43.1%	0.08
Married and living separated from spouse	6	40.0%	9	60.0%	
Divorced	27	40.9%	39	59.1%	
Widowed	70	58.8%	49	41.2%	
Single	18	54.6%	15	45.4%	
Employment status					
Employed	85	38.3%	137	61.7%	<0.001
Retired	390	63.7%	222	36.3%	
Other: not employed	65	47.8%	71	52.2%	
Monthly net equivalence income (€)	1477.2	670.3	1696.0	927.7	<0.001
Meaning in life (from 1 to 5; higher values correspond to higher meaningfulness)	4.2	0.7	4.2	0.8	0.88
Self-rated health (ranging from 1 = very good to 5 = very bad)	2.5	0.7	2.3	0.7	<0.01
Number of physical illnesses (from 0 to 11)	2.8	1.7	2.2	1.7	<0.001
Absence of depression	475	55.5%	381	44.5%	0.58
Physical functioning (ranging from 0 (worst) to 100 (best))	83.9	19.9	87.3	18.6	<0.01
Loneliness (from 1 to 4; higher values correspond to higher loneliness)	1.6	0.5	1.7	0.5	0.03

Notes: *p*-values are based on chi square tests or *t*-tests, as appropriate.

**Table 2:** Determinants of flu vaccination in the past 12 months (0 = no; 1 = yes). Results of multiple logistic regressions.

Independent Variables	Flu Vaccinations
Lonely (from 1 to 4; higher values correspond to higher loneliness)	0.71 *
	(0.51–0.97)
Sex (ref.: male)	1.17
	(0.85–1.61)
Meaning in life (from 1 to 5; higher values correspond to higher meaningfulness)	1.05
	(0.85–1.29)
Age	1.00
	(0.97–1.03)
Marital status	
Married, living separated from spouse (ref.: married, living together with spouse)	0.65
	(0.20–2.05)
Divorced	0.55 +
	(0.30–1.01)
Widowed	0.77
	(0.47–1.27)
Single	1.18
	(0.48–2.90)
Employment status	
Retired (ref.: employed)	1.99 **
	(1.23–3.23)
Other: not employed	1.11
	(0.66–1.86)
Log household net equivalent income	0.62 **
	(0.44–0.88)
Depression (ref.: absence of depression)	0.72
	(0.38–1.37)
Self-rated health (ranging from 1 = very good to 5 = very bad)	1.16
	(0.90–1.48)
Physical functioning (ranging from 0 (worst) to 100 (best))	1.00
	(0.99–1.01)
Number of physical illnesses (ranging from 0 to 11)	1.20 ***
	(1.09–1.32)
Constant	18.75
	(0.46–761.03)
Pseudo R²	0.07
	
Observations	785

Odds ratios were reported; 95% confidence intervals in parentheses; *** *p* < 0.001, ** *p* < 0.01, * *p* < 0.05, + *p* < 0.10; due to missing values, 785 observations remain in the regression analysis.
